# Unsupervised Quality Estimation Model for English to German Translation and Its Application in Extensive Supervised Evaluation

**DOI:** 10.1155/2014/760301

**Published:** 2014-04-28

**Authors:** Aaron L.-F. Han, Derek F. Wong, Lidia S. Chao, Liangye He, Yi Lu

**Affiliations:** Natural Language Processing & Portuguese-Chinese Machine Translation Laboratory, Department of Computer and Information Science, University of Macau, Macau

## Abstract

With the rapid development of machine translation (MT), the MT evaluation becomes very important to timely tell us whether the MT system makes any progress. The conventional MT evaluation methods tend to calculate the similarity between hypothesis translations offered by automatic translation systems and reference translations offered by professional translators. There are several weaknesses in existing evaluation metrics. Firstly, the designed incomprehensive factors result in language-bias problem, which means they perform well on some special language pairs but weak on other language pairs. Secondly, they tend to use no linguistic features or too many linguistic features, of which no usage of linguistic feature draws a lot of criticism from the linguists and too many linguistic features make the model weak in repeatability. Thirdly, the employed reference translations are very expensive and sometimes not available in the practice. In this paper, the authors propose an unsupervised MT evaluation metric using universal part-of-speech tagset without relying on reference translations. The authors also explore the performances of the designed metric on traditional supervised evaluation tasks. Both the supervised and unsupervised experiments show that the designed methods yield higher correlation scores with human judgments.

## 1. Introduction

The research about machine translation (MT) can be traced back to fifty years ago [1] and people benefit much from it about the information exchange with the rapid development of the computer technology. Many MT methods and automatic MT systems were proposed in the past years [2–4]. Traditionally, people use the human evaluation approaches for the quality estimation of MT systems, such as the adequacy and fluency criteria. However, the human evaluation is expensive and time consuming. This leads to the appearance of the automatic evaluation metrics, which give quick and cheap evaluation for MT systems. Furthermore, the automatic evaluation metrics can be used to tune the MT systems for better output quality. The commonly used automatic evaluation metrics include BLEU [5], METEOR [6], TER [7], AMBER [8], and so forth. However, most of the automatic MT evaluation metrics are reference aware, which means they tend to employ different approaches to calculate the closeness between the hypothesis translations offered by MT systems and the reference translations provided by professional translators. There are some weaknesses in the conventional reference-aware methods: (1) how many reference translations are enough to avoid the evaluation bias since that reference translations usually cannot cover all the reasonable expressions? (2) The reference translation is also expensive and sometimes not approachable in practice. This paper will propose an automatic evaluation approach for English-to-German translation by calculating the similarity between source and hypothesis translations without using of reference translation. Furthermore, the potential usage of the proposed evaluation algorithms in the traditional reference-aware MT evaluation tasks will also be explored.

## 2. Traditional MT Evaluations

### 2.1. BLEU Metric

The commonly used BLEU (bilingual evaluation understudy) metric [5] is designed as automated substitute to skilled human judges when there is need for quick or frequent MT evaluations:

(1) $$\begin{array}{c} \text{BLEU}=\text{BP}\times \exp\left(\overset{N}{\underset{n=1}{\sum }}w_{n}logP_{n}\right),\\ P_{n}=\frac{\sum_{C\in \{\text{Candidates}\}}\sum_{n-\text{gram}\in C}\text{Coun}{\text{t}}_{\text{clip}}\left(n-\text{gram}\right)}{\sum_{C^{\prime }\in \{\text{Candidates}\}}\sum_{n-\text{gra}{\text{m}}^{\prime }\in C^{\prime }}\text{Count}\left(n-\text{gra}{\text{m}}^{\prime }\right)}, \end{array}$$

where *P*_*n*_ means modified *n*-gram precision on a multisentence test set, which is for the entire test corpus. It first computes the *n*-gram matches sentence by sentence and then adds the clipped *n*-gram counts for all the candidate sentences and divides by the number of candidate *n*-gram in the test corpus. Consider

(2) $$\begin{array}{c} \text{BP}=\{\begin{array}{cc} e^{1-r/c} & \text{if}\;\;c\leq r\\ 1 & \text{if}\;\;c>r, \end{array} \end{array}$$

where BP is the sentence brevity penalty for short sentences, *r* is the effective reference sentence length (the closest reference sentence length with the candidate sentence), and *c* is the length of candidate sentence. Generally, *N* is selected as 4, and uniform weight *w*_*n*_ is assigned as 1/*N*. Thus we could get the deduction as follows:

(3) BLEUbaseline=BP×exp⁡⁡(∑n=1N1Nlog⁡Pn)=BP×exp⁡⁡(1Nlog⁡∏n=1NPn)=BP×exp⁡⁡(log⁡(∏n=1NPn)1/N)=BP×(∏n=1NPn)1/N=BP×∏n=1NPnN.

This shows that BLEU reflects the geometric mean of *n*-gram precision values multiplied by brevity penalty. As a contrast and simplified version, Zhang et al. [9] proposes modified BLEU metric M_BLEU using the arithmetic mean of the *n*-gram precision:

(4) $$\begin{array}{c} \text{M}\_\text{BLEU}=\text{BP}\times \overset{N}{\underset{n=1}{\sum }}w_{n}P_{n}. \end{array}$$

The weaknesses of BLEU series are that they focus on the usage of incomprehensive factors, precision scores only; they do not use any linguistic features, only utilizing the surface words.

### 2.2. TER Metric

TER [7] means translation edit rate, which is designed at sentence level to calculate the amount of work needed to correct the hypothesis translation according to the closest reference translation (assuming there are several reference translations):

(5) $$\begin{array}{c} \text{TER}=\frac{\#\;\text{of}\;\text{edits}}{\text{average}\;\#\;\text{of}\;\text{reference}\;\text{words}}. \end{array}$$

The edit categories include the insertion, deletion, substitution of single words, and the shifts of word chunks. TER uses the strict matching of words and word order, for example, miscapitalization is also counted as an edit. The weakness of TER is that it gives an overestimate of the actual translation error rate since that it requests accurate matching between the reference and hypothesis sentence. To address this problem, they proposed the human-targeted TER (HTER) to consider the semantic equivalence, which is achieved by employing human annotators to generate a new targeted reference. However, HTER is very expensive due to that it requires around 3 to 7 minutes per sentence for a human to annotate, which means that it is more like a human judgment metric instead of an automatic one.

### 2.3. METEOR Metric

METEOR [6] metric conducts a complicated matching, considering stems, synonyms, and paraphrases. Consider

(6) $$\begin{array}{c} \text{Score}=F_{\text{mean}}\times \left(1-\text{Penalty}\right),\\ \text{Penalty}=0.5\times {\left(\frac{\#\text{chunks}}{\#\text{unigrams}\_\text{matched}}\right)}^{3},\\ F_{\text{mean}}=\frac{10PR}{R+9P}, \end{array}$$

where #chunks means the number of matched chunks between reference and hypothesis sentence, and #unigrams_matched is the number of matched words. It puts more weight on recall (*R*) compared with precision (*P*). The matching process involves computationally expensive word alignment due to the external tools for stemming or synonym matches. The advanced version of METEOR is introduced in [10].

### 2.4. AMBER Metric

AMBER [8] declares a modified version of BLEU. It attaches more kinds of penalty coefficients, combining the *n*-gram precision and recall with the arithmetic average of *F*-measure (harmonic mean of precision and recall with the equal weight). It provides eight kinds of preparations on the corpus including whether the words are tokenized or not, extracting the stem, prefix and suffix on the words, and splitting the words into several parts with different ratios. Advanced version of AMBER was introduced in [11]. Other related works about traditional reference-aware MT evaluation metrics can be referred to in the papers [12, 13], our previous works [14, 15], and so forth.

## 3. Related Works

As mentioned previously, the traditional evaluation metrics tend to estimate quality of the automatic MT output by measuring its closeness with the reference translations. To address this problem, some researchers design the unsupervised MT evaluation approaches without using reference translations, which is also called quality estimation of MT. For example, Han et al. design an unsupervised MT evaluation for French and English translation using their developed universal phrase tagset [16] and explore the performances of machine learning algorithms, for example, conditional random fields, support vector machine and naïve Bayes in the word-level quality estimation task of English to Spanish translation without using golden references [17]. Gamon et al. [18] conduct a research about reference-free MT evaluation approaches also at sentence level, which utilizes the linear and nonlinear combinations of language model and SVM classifier to find the badly translated sentences. Using the regression learning and a set of indicators of fluency and adequacy as pseudoreferences, Albrecht and Hwa [19] present an unsupervised MT evaluation work at sentence level performance. Employing the confidence estimation features and a learning mechanism trained on human annotations, Specia and Giménez [20] develop some quality estimation models, which are biased by difficulty level of the input segment. The issues between the traditional supervised MT evaluations and the latest unsupervised MT evaluations are discussed in the work of [21]. The quality estimation addresses this problem by evaluating the quality of translations as a prediction task and the features are usually extracted from the source sentences and target (translated) sentences. Using the IBM model one and the information of morphemes, lexicon probabilities, part-of-speech, and so forth, Popović et al. [22] also introduces an unsupervised evaluation method and show that the most promising setting comes from the IBM-1 scores calculated on morphemes and POS-4gram. Mehdad et al. [23] use the cross-lingual textual entailment to push semantics into the MT evaluation without using reference translations, which mainly focuses on the adequacy estimation. Avramidis [24] performs an automatic sentence-level ranking of multiple machine translations using the features of verbs, nouns, sentences, subordinate clauses, and punctuation occurrences to derive the adequacy information. Other related works that introduce the unsupervised MT evaluations include [25, 26].

## 4. Designed Approach

To reduce the expensive reference translations provided by human labor and some external resources such as synonyms, this work employs the universal part-of-speech (POS) tagset containing 12 universal tags proposed by [27]. A part-of-speech is a word class, a lexical class, or a lexical category, which is a linguistic category of words (or lexical items). It is generally defined by the syntactic or morphological behavior of the lexical item in question.

For a simple example, “there is a big bag” and “there is a large bag” could be the same expression, “big” and “large” having the same POS as adjective. To try this potential approach, we conduct the evaluation on the POS of the words from the source language and the target language. The source language is used as pseudoreference. We will also test this method by calculating the correlation coefficient of this approach with the human judgments in the experiment. Petrov et al. [27] describe that the English PennTreebank [28] has 45 tags and German Negra [29] has 54 tags. However, in the mapping table they offer 49 tags for the German Negra Treebank. This paper makes a test on the Berkeley parser [30] for German (trained on the German Negra) parsing a German corpus from the workshop of machine translation (WMT) 2012 [25] and finds that there are indeed other POS tags that are not included in the mapped 49 tags. So, firstly this paper conducts a complementary mapping work for German Negra POS tagset and extends the mapped POS tags to 57 tags.

### 4.1. Complementary POS Mapping

The parsing test result of WMT 2012 German corpus shows that the omissive German POS tags in the mapping table include “PWAV, PROAV, PIDAT, PWAT, PWS, PRF, $∗LRB∗, and ∗TN∗,” of which “∗TN∗” is a formal style (*N* is replaced in practical parsing results by the integer number such as 1, 2, etc.).

This paper classifies the omissive German POS tags according to the English POS tagset classification since that the English PennTreebank 45 POS tags are completely mapped by the universal POS tagset, as shown in Table 1.

- The German POS “PWAV” has a similar function to English POS “WRB” which means wh-adverb labeling the German word such as während (while), wobei (where), wann (when), and so forth. The German POS “PROAV” has a similar function to English POS “RB” which means adverb labeling the German word Dadurch (thereby), dabei (there), and so forth. So this paper classifies “PWAV” and “PROAV” into the ADV (adverb) category in the 12 universal POS tags.
- The German POS “PIDAT” has a similar function to English POS “PDT” which means predeterminer labeling the German word jedem (each), beide (both), meisten (most), and so forth. The German POS “PWAT” has a similar function to English POS “WDT” which means wh-determiner labeling the German word welche (which), welcher (which), and so forth. So “PIDAT” and “PWAT” are classified into the DET (determiner) category in the universal POS tags.
- The German POS “PWS” has a similar function to English POS “WP” which means wh-pronoun labeling the German word was (what), wer (who), and so forth. So “PWS” is classified into the PRON (pronoun) category in the universal POS tags.
- The German POS “PRF” has a similar function to English POS “RP” and “TO”, which means particle and to, respectively, labeling the German word sich (itself). So this paper classifies “PRF” into the PRT (a less clear case for particle, possessive, and to) category.
- The German POS “∗TN∗” and “$∗LRB∗” are classified into the punctuation category since that they are only used to label the German punctuations such as dash, bracket, and so forth. After all of the complementary mapping, the universal POS tagset alignment for German Negra Treebank is shown in Table 1 with the boldface POS as the added ones.

### 4.2. Calculation Algorithms

The designed calculation algorithms of this paper are LEPOR series. First, we introduce the nLEPOR model, *n*-gram based quality estimation metric for machine translation with augmented factor of enhanced length penalty, precision, position difference penalty, and recall. We will introduce the subfactors in the formula step by step:

(7) nLEPOR=LP×NPosPenal×exp⁡(∑n=1Nwnlog⁡H(αRn,βPn))LP={e1−s/cif  c<s1if  c=se1−c/sif  c>s.

In the formula, LP means enhanced sentence length penalty that is designed for both the shorter or longer translated sentences (hypothesis) compared with the source sentence. This approach is different with BLEU metric which assigns penalty for the shorter sentence compared with the human reference translation. Parameters *c* and *s* specify the length of candidate sentence (hypothesis) and source sentence, respectively.

The variable NPosPenal means *n*-gram position difference penalty that is designed for the different order of successful matched POS in source and hypothesis sentence. The position difference factor has been proved to be helpful for the MT evaluation in the research work of [13]. The alignment direction is from hypothesis to the source sentence with the algorithm shown in Algorithm 1. This paper employs the *n*-gram method into the matching process, which means that the potential POS candidate will be assigned higher priority if it has neighbor matching. The nearest matching will be accepted as a backup choice if there are both neighbor matching or there is no other matched POS around the potential pairs. In Algorithm 1, assuming that *p*_*x*_ represents the current universal POS in hypothesis sentence, *p*_*x*+*k*_ means the *k*th POS to the previous (*k* < 0) or following (*k* > 0) position. It is the similar approach for the source sentence. Consider

(8) $$\begin{array}{c} \text{NPosPenal}=e^{-\text{NPD}},\\ \text{NPD}=\frac{1}{\text{Lengt}{\text{h}}_{\text{hyp}}}\overset{\text{Lengt}{\text{h}}_{\text{hyp}}}{\underset{i=1}{\sum }}\left|\text{P}{\text{D}}_{i}\right|,\\ \left|{\text{PD}}_{i}\right|=\left|{\text{MatchPosN}}_{\text{hyp}}-{\text{MatchPosN}}_{\text{src}}\right|. \end{array}$$

**Figure 1:**
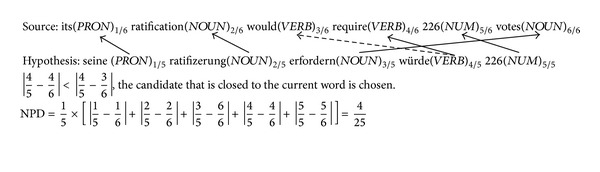
Universal POS alignment and NPD calculation example.

The parameter Length_hyp_ means the length of hypothesis sentence, MatchPosN_hyp_, and MatchPosN_src_ as the matched POS position number in hypothesis and source sentence, respectively. See Figure 1 as an example.

The parameter *w*_*n*_ is designed to adjust the weights of different *n*-gram performances such as unigram, bigram, trigram, four-gram, and so forth, which is different with the weight assignment in BLEU where each weight is equal to 1/*N*. In our model, higher weight value is designed for the high level *n*-gram. Consider the following:

(9) $$\begin{array}{c} w_{n}=\frac{n}{\sum_{i=1}^{N}i}=\frac{n}{1+2+\cdots +N},\\ H\left(\alpha R_{n},\beta P_{n}\right)=\frac{\left(\alpha +\beta \right)}{\left(\alpha /R_{n}+\beta /P_{n}\right)}. \end{array}$$

**Figure 2:**
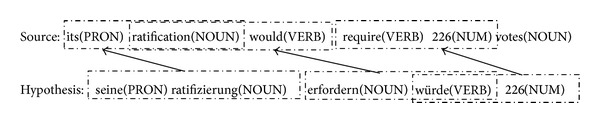
Universal POS chunk matching example for bigram precision and recall.

The factor *H*(*αR*_*n*_, *βP*_*n*_) is the mathematical harmonic mean of *n*-gram precision (*P*_*n*_) and *n*-gram recall (*R*_*n*_) in (10), where #*n*gram_matched_ represents the number of matched *n*-gram chunks. The *n*-gram precision (and recall) is calculated on sentence level not corpus-level used in BLEU (*P*_*n*_). Let us see the example in Figure 1 again for the explanation of bigram precision *P*_2_ and bigram recall *R*_2_. The number of bigram chunks in hypothesis is 4 (PRON NOUN, NOUN NOUN, NOUN VERB, and VERB NUM), the number of bigram chunks in source is 5 (PRON NOUN, NOUN VERB, VERB VERB, VERB NUM, and NUM NOUN), and the number of matched bigram chunk is 3 (PRON NOUN, NOUN VERB, and VERB NUM) as shown in Figure 2. So the value of *P*_2_ and *R*_2_ equals 3/4 and 3/5, respectively.

(10) $$\begin{array}{c} P_{n}=\frac{\#{n\text{gram}}_{\text{matched}\;}}{\#n\text{gram}\;\text{chunks}\;\text{in}\;\text{hypothesis}},\\ R_{n}=\frac{\#{n\text{gram}}_{\text{matched}}}{\#n\text{gram}\;\text{chunks}\;\text{in}\;\text{source}}. \end{array}$$

### 4.3. System-Level Metric

We design two approaches for document-level calculation for the proposed algorithms:

(11) nLEPORA−=1SentNum∑i=1SentNumNLEPORi,nLEPORB−=LP−×PosPenalty−×exp⁡(∑n=1Nwnlog⁡H(αRn,βPn))−.

The document-level $\overset{-}{\text{nLEPO}{\text{R}}_{A}}$ is calculated as the arithmetic mean value of each sentence-level score in the document. On the other hand, the document-level $\overset{-}{\text{nLEPO}{\text{R}}_{B}}$ is calculated as the product of three document-level variables, and the document-level variable value is the corresponding arithmetic mean score of each sentence.

## 5. Usage in Traditional Evaluation

We introduce the potential usage of the proposed evaluation algorithms in traditional supervised (reference-aware) MT evaluation methods. In the unsupervised design, the nLEPOR metric is measured on the source and target POS sequences instead of the surface words. In the exploration of its usage in the traditional supervised MT evaluations, we measure the nLEPOR score on the target translations (system outputs) and reference translations, that is, measuring on the surface words. The pseudoreference (source language) is replaced with real reference here. The performance of simplified variant nLEPOR_*A*_ will be tested using the reference translations.

## 6. Evaluating the Evaluation Method

The conventional method to evaluate the quality of different automatic MT evaluation metrics is to calculate their correlation scores with human judgments. The Spearman rank correlation score (*r*_*s*_) and Pearson correlation score *ρ* are commonly used by the annual workshop of statistical machine translation (WMT) of Association for Computational Linguistics (ACL) [25, 31, 32]. Assuming that $\overset{⃑}{X}=\{x_{1},x_{2},\ldots ,x_{n}\}$ and $\overset{⃑}{Y}=\{y_{1},y_{2},\ldots ,y_{n}\}$ are two rank sequences and *n* is the number of variables, when there are no ties, Spearman rank correlation coefficient is calculated as

(12) $$\begin{array}{c} r_{s_{\emptyset (XY)}}=1-\frac{6\sum_{i=1}^{n}d_{i}^{2}}{n\left(n^{2}-1\right)}, \end{array}$$

where *d*_*i*_ is the difference value (*D*-value) between the two coordinate rank variables (*x*_*i*_ − *y*_*i*_).

Secondly, the Pearson correlation coefficient information is introduced as below. Given a sample of paired data (*X*, *Y*) as (*x*_*i*_, *y*_*i*_),  *i* = 1 to *n*, the Pearson correlation coefficient is

(13) $$\begin{array}{c} \rho_{XY}=\frac{\sum_{i=1}^{n}\left(x_{i}-\mu_{x}\right)\left(y_{i}-\mu_{y}\right)}{\sqrt{\sum_{i=1}^{n}{\left(x_{i}-\mu_{x}\right)}^{2}}\sqrt{\sum_{i=1}^{n}{\left(y_{i}-\mu_{y}\right)}^{2}\;}}, \end{array}$$

where *μ*_*x*_ and *μ*_*y*_ specify the arithmetical means of discrete random variable *X* and *Y*, respectively.

## 7. Experiments

### 7.1. Unsupervised Performances

In the unsupervised MT evaluation, this paper uses the English-to-German machine translation corpora (produced by around twenty English-to-German MT systems) from ACL-SIGMT (http://www.sigmt.org/), which makes the annual workshop corpora public available for further research purpose. Each document contains 3,003 sentences of source English or translated German. To avoid the over fitting problem, the WMT2011 (http://www.statmt.org/wmt11/) corpora are used as training data and WMT2012 (http://www.statmt.org/wmt12/) corpora are used for the testing. This paper conducts the experiments on the simplified version (unigram precision and recall) of the metric.

Table 2 shows the system-level Spearman rank correlation scores of nLEPOR with human judgments trained on the WMT2011 data, as compared to several state-of-the-art reference-aware automatic evaluation metrics including BLEU, METEOR, and AMBER.

In the training period, the parameter values of *α* (weight on recall) and *β* (weight on precision) are tuned to 1 and 9, respectively, which is different with the reference-aware metric METEOR (more weight on recall). Bigram is selected for the *n*-gram universal POS alignment period. The correlation scores in Table 2 show that the proposed evaluation approaches of this paper have achieved higher score (0.63 and 0.60, resp.) in training period than the other evaluation metrics (with the score 0.53, 0.44, and 0.30, resp.) apparently even though the compared metrics are reference aware.

Testing result of the proposed evaluation approaches on the WMT2012 corpora is shown in Table 3 with the same parameter values obtained as in training, also compared with the state-of-the-art evaluation metrics. The correlation scores in Table 3 show the same rank results with Table 2, METEOR achieving the lowest score (0.18), AMBER (0.25) achieving score higher than BLEU (0.22), and NLEPOR family yielding the highest correlation coefficient (0.34 and 0.33, resp.) with human judgments as previous. The test results show the robustness of the proposed evaluation approaches. The experiments also show that the latest proposed metrics (e.g., AMBER) achieve higher correlation score than the earlier ones (e.g., BLEU).

### 7.2. Supervised Performances

As mentioned previously, to explore the performance of the designed algorithm nLEPOR_*A*_ in the traditional reference-aware MT evaluation track, we also use the WMT11 corpora as training data and WMT12 corpora as testing data to avoid the overfitting phenomenon. The number of participated MT systems that offer the output translations is shown in Table 5 for each language pair. In the training period, the tuned values of *α* and *β* are 9 and 1, respectively, for all language pairs except for (*α* = 1, *β* = 9) for CS-EN. The training results on WMT11 eight corpora including English-to-other (CS-Czech, DE-German, ES-Spanish, FR-French), and other-to-English are shown in Table 4. The aim of the training stage is to achieve higher correlation with human judgments. The experiments on WMT11 corpora show that nLEPOR_*A*_ yields the best correlation scores on the language pairs of CS-EN, ES-EN, EN-CS, and EN-ES, which contributes to the highest average score 0.77 on all the eight language pairs. The testing results on WMT12 corpora show that nLEPOR_*A*_ yields the highest correlation score with human judgments on CS-EN (0.89) and EN-ES (0.45) language pairs, and the highest average correlation scores on other-to-English (0.85), English-to-other (0.58), and all the eight corpora (0.71) (Table 6).

### 7.3. Enhanced Model and the Performances

In the previous sections, we have introduced the *n*-gram based metric nLEPOR and its performances in both supervised and unsupervised cases. In this section we will introduce an enhanced version of the proposed metric, which is called as hLEPOR (harmonic mean of enhanced length penalty, precision, *n*-gram position difference penalty, and recall). There are two contributions of this enhanced model. Firstly, it assigns different weights to three subfactors, which are tunable according to different language pairs. This property can help to address the language-bias problem existing in many current automatic evaluation metrics. Secondly, it is designed to combine the performances on words and POS together, and the final score is the combination of them:

(14) $$\begin{array}{c} \text{hLEPOR}=\frac{w_{\text{LP}}+w_{\text{NPosPenal}}+w_{\text{HPR}}}{w_{\text{LP}}/\text{LP}+w_{\text{NPosPenal}}/\text{NPosPenal}+w_{\text{HPR}}/\text{HPR}}, \end{array}$$

where HPR is the harmonic mean of precision and recall as mentioned above *H*(*αR*_*n*_, *βP*_*n*_). Consider

(15) hLEPORfinal=1whw+whp ×(whwhLEPORword+whphLEPORPOS).

Firstly, we calculate the hLEPOR score on surface words hLEPOR_word_, that is, the closeness of the hypothesis translation and the reference translation. Then we calculate the hLEPOR score on the extracted POS sequences hLEPOR_POS_, that is, the closeness of the corresponding POS tags between hypothesis sentence and reference sentence. The final score hLEPOR_final_ is the combination of the two subscores hLEPOR_word_ and hLEPOR_POS_.

We introduce the performances of nLEPOR and hLEPOR in the WMT13 (http://www.statmt.org/wmt13/) shared evaluation tasks below. Both of the two metrics are trained on WMT11 corpora. The tuned parameters of hLEPOR are shown in Table 7, using default values for EN-RU and RU-EN. The number of MT systems for each language pair in WMT13 is shown in Table 8. There is a new language Russian in WMT13, which leads to the increasing of total number of corpora into ten. Due to the fact that there is no Russian language in the past WMT shared tasks, for EN-RU and RU-EN corpora, we assign the default values *α* = 1 and *β* = 1 in nLEPOR.

In the WMT13 shared tasks, both Pearson correlation coefficient and the Spearman rank correlation coefficient are used as the evaluation criteria. So we list the official results in Tables 9 and 10, respectively, by using Spearman rank correlation and Pearson correlation criteria.

Using the Spearman rank correlation coefficient, the experiment results on WMT13 in Table 9 show that hLEPOR yields the highest correlation scores on EN-DE (0.90), EN-RU (0.85), and the highest average correlation score (0.85) on five English-to-other corpora; nLEPOR_*A*_ yields the highest correlation scores on EN-DE (0.90), EN-ES (0.85), and EN-FR (0.92) and the second highest average correlation score (0.84) on five English-to-other corpora.

Using the Pearson correlation coefficient, the experiment results on WMT13 in Table 10 show that hLEPOR yields the highest correlation scores on EN-DE (0.94), EN-ES (0.91), and EN-RU (0.77) and the highest average correlation score (0.86) on five English-to-other corpora; nLEPOR_*A*_ yields the highest correlation scores on EN-ES (0.82) and EN-FR (0.92) and the second highest average correlation score (0.85) on five English-to-other corpora.

On the other hand, METEOR yields the best performances on other-to-English translation evaluation direction. This is due to the fact that METEOR employs many external materials including stemming, synonyms vocabulary, paraphrasing resources, and so forth. However, to make the evaluation model concise, our method nLEPOR only uses the surface words and hLEPOR only uses the combination of surface words and POS sequences. This shows the advantages of our designed methods, that is, concise linguistic feature.

As mentioned previously, the language-bias problem is presented in the evaluation results of BLEU metric. BLEU yields the highest Spearman rank correlation score 0.99 on FR-EN; however it never achieves the highest average correlation score on any translation direction, due to the very low correlation scores on RU-EN, EN-DE, EN-ES, and EN-RU corpora. Our metrics LEPOR series address the language-bias problem by using augmented factors and tunable parameters.

The evaluation results in Tables 9 and 10 show that the evaluation on the language pairs with English as the source language (English-to-other) is the main challenge at system-level performance. Fortunately, the designed evaluation methods of this paper have made some contributions on this translation evaluation direction.

## 8. Discussion

The Spearman rank correlation coefficient is commonly used in the WMT shared tasks as the special case of Pearson correlation coefficient applied to ranks. However, there is some information that cannot be reflected by using Spearman rank correlation coefficient instead of Pearson correlation coefficient. For example, let us assume there are three automatic MT systems $\vec{M}=\left\{M_{1},M_{2},M_{3}\right\}$ and two automatic MT evaluation systems MTE_*A*_ and MTE_*B*_. The evaluation scores of the two evaluation systems on the three MT systems are $\vec{\text{MT}{\text{E}}_{A}}=\left\{0.90,0.35,0.40\right\}$ and $\vec{\text{MT}{\text{E}}_{B}}=\left\{0.46,0.35,0.42\right\}$, respectively. Using the Spearman rank correlation coefficient, the two vectors will be first converted into $\overset{ˇ}{\vec{\text{MT}{\text{E}}_{A}}}=\left\{1,3,2\right\}$ and $\overset{ˇ}{\vec{\text{MT}{\text{E}}_{B}}}=\left\{1,3,2\right\}$, respectively, then their correlation with human rank results (human judgments) will be measured. Thus, the two evaluation metrics will yield the same Spearman rank correlation score with human judgments no matter what the manual evaluation results will be. However, the evaluation systems MTE_*A*_ and MTE_*B*_ actually tell different results on the quality of the three MT systems. The evaluation system MTE_*A*_ gives very high score 0.90 on *M*_1_ system and similar low scores 0.35 and 0.40, respectively, on *M*_2_ and *M*_3_ systems. On the other hand, the evaluation system MTE_*B*_ yields similar scores 0.46, 0.35, and 0.42, respectively, on the three MT systems, which means that all the three automatic MT systems have low translation quality. Using the Spearman rank correlation coefficient, the above important information is lost.

On the other hand, the Pearson correlation coefficient uses the absolute scores yielded by the automatic MT evaluation systems as shown in (13), without the preconverting into rank values.

## 9. Conclusions and Future Works

To avoid the usage of expensive reference translations, this paper designs a novel unsupervised MT evaluation model for English to German translation by employing augmented factors and universal POS tagset. Furthermore, the proposed unsupervised model yields higher correlation score with human judgments as compared to the reference-aware metrics METEOR, BLEU, and AMBER.

The application of the designed algorithms to the traditional supervised evaluation tasks is also explored. To address the language-bias problem in most of the existing metrics, tunable parameters are assigned to different subfactors. The experiment on WMT11 and WMT12 corpora shows that our designed algorithms yields the highest average correlation score on eight language pairs as compared to the state-of-the-art reference-aware metrics METEOR, BLEU, and TER.

On the other hand, to address the linguistic-extreme problem (no linguistic information or too many linguistic features), our method utilizes the optimized linguistic feature POS sequence, in addition to the surface words, to make the model concise and easy to repeat.

Last, this paper also makes a contribution to the complementary POS tagset mapping between German and English in the light of 12 universal tags.

The developed algorithms in this paper are freely available for research purpose (https://github.com/aaronlifenghan/aaron-project-lepor). In the future works, to test the robustness of the designed algorithms and models, we will seek more language pairs, such as the Asian languages Chinese, Korean, and Japanese, to conduct the experiments, in addition to the official European languages offered by SIGMT association. Secondly, the experiments using multireferences will be considered. Thirdly, how to handle the MT evaluation from the aspect of semantic similarity will be further explored.

## Figures and Tables

**Algorithm 1:**
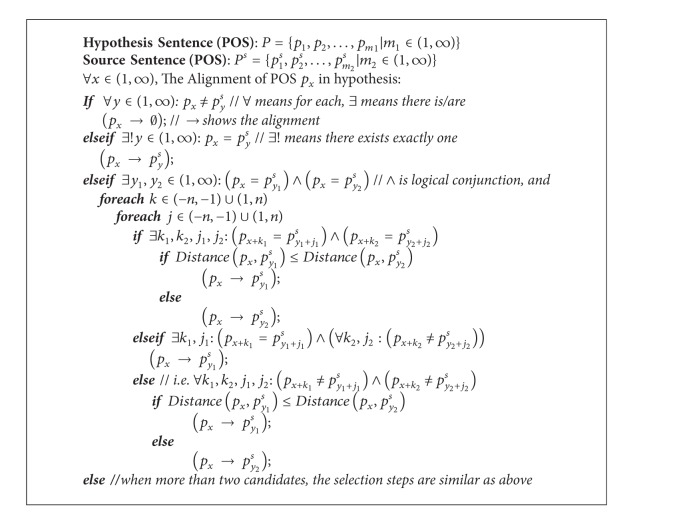
The POS alignment algorithm from hypothesis to source sentence based on *n*-gram.

**Table 1:** Complementary German POS (as bold) mappings for Universal POS tagset.

Language	ADJ	ADP	ADV	CONJ	DET	NOUN	NUM	PRON	PRT	VERB	X	.
English Penn Treebank [28]	JJ JJR JJS	IN	RB RBR RBS WRB	CC	DT EX PDT WDT	NN NNP NNPS NNS	CD	PRP PRP$ WP WP$	POS RP TO	MD VB VBD VBG VBN VBP VBZ	FW LS SYM UH	# $ ” . : -LRB- -RRB-

German Negra Treebank [29]	ADJA ADJD	APPO APPR APPRART APZR	ADV **PWAV** **PROAV**	KOKOM KON KOUI KOUS	ART **PIDAT** **PWAT**	NE NN NNE	CARD	PDAT PDS PIAT PIS PPER PPOSAT PPOSS PRELAT PRELS **PWS**	PTKA PTKANT PTKNEG PTKVZ PTKZU **PRF**	VAFIN VAIMP VAINF VAPP VMFIN VMINF VMPP VVFIN VVIMP VVINF VVIZU VVPP	FM ITJ TRUNC XY	$( $, $. **∗TN∗** **$∗LRB∗**

**Table 2:** Performances on the WMT11 training corpora.

Metrics	UseReference?	rs
AMBER	Yes	0.53
BLEU	Yes	0.44
METEOR	Yes	0.30
$\overset{-}{\text{nLEPO}{\text{R}}_{A}}$	**No**	**0.63**
$\overset{-}{\text{nLEPO}{\text{R}}_{B}}$	**No**	0.60

Bold fonts mean the best performance.

**Table 3:** Performances on the WMT12 testing corpora.

Metrics	UseReference?	rs
AMBER	Yes	0.25
BLEU	Yes	0.22
METEOR	Yes	0.18
$\overset{-}{\text{nLEPO}{\text{R}}_{A}}$	**No**	**0.34**
$\overset{-}{\text{nLEPO}{\text{R}}_{B}}$	**No**	0.33

Bold fonts mean the best performance.

**Table 4:** The performances on WMT11 training corpora using Spearman correlation.

System	Correlation score with human judgments										
	Other-to-English					English-to-other					Mean
	CS-EN	DE-EN	ES-EN	FR-EN	Mean	EN-CS	EN-DE	EN-ES	EN-FR	Mean	
nLEPOR_*A*_	**0.95**	0.61	**0.96**	0.88	0.85	**0.68**	0.35	**0.89**	0.83	0.69	**0.77**
METEOR	0.91	**0.71**	0.88	**0.93**	**0.86**	0.65	0.30	0.74	0.85	0.64	0.75
BLEU	0.88	0.48	0.90	0.85	0.78	0.65	**0.44**	0.87	**0.86**	**0.71**	0.74
TER	0.83	0.33	0.89	0.77	0.71	0.50	0.12	0.81	0.84	0.57	0.64

Bold fonts mean the best performance.

**Table 5:** The number of effective MT systems in WMT11 and WMT12.

Year	Number of evaluated MT systems							
	Other-to-English				English-to-other			
	CS-EN	DE-EN	ES-EN	FR-EN	EN-CS	EN-DE	EN-ES	EN-FR
WMT11	8	20	15	18	10	22	15	22
WMT12	6	16	12	15	13	15	11	15

**Table 6:** The performances on WMT12 testing corpora using Spearman correlation.

System	Correlation score with human judgments										
	Other-to-English					English-to-other					Mean
	CS-EN	DE-EN	ES-EN	FR-EN	Mean	EN-CS	EN-DE	EN-ES	EN-FR	Mean	
nLEPOR_*A*_	**0.89**	0.77	0.91	0.81	**0.85**	0.75	0.34	**0.45**	0.77	**0.58**	**0.71**
METEOR	0.66	**0.89**	**0.95**	**0.84**	0.84	0.73	0.18	**0.45**	**0.82**	0.55	0.69
BLEU	**0.89**	0.67	0.87	0.81	0.81	**0.80**	0.22	0.40	0.71	0.53	0.67
TER	**0.89**	0.62	0.92	0.82	0.81	0.69	**0.41**	**0.45**	0.66	0.55	0.68

Bold fonts mean the best performance.

**Table 7:** The tuned weight values in hLEPOR.

Ratio	Other-to-English					English-to-other				
	CZ-EN	DE-EN	ES-EN	FR-EN	RU-EN	EN-CZ	EN-DE	EN-ES	EN-FR	EN-RU
HPR : LP : NPosPenal (word)	7 : 2 : 1	3 : 2 : 1	7 : 2 : 1	3 : 2 : 1	3 : 2 : 1	7 : 2 : 1	1 : 3 : 7	3 : 2 : 1	3 : 2 : 1	3 : 2 : 1
HPR : LP : NPosPenal (POS)	NA	3 : 2 : 1	NA	3 : 2 : 1	3 : 2 : 1	7 : 2 : 1	7 : 2 : 1	NA	3 : 2 : 1	3 : 2 : 1
*α* : *β* (word)	1 : 9	9 : 1	1 : 9	9 : 1	1 : 1	9 : 1	9 : 1	9 : 1	9 : 1	1 : 1
*α* : *β* (POS)	NA	9 : 1	NA	9 : 1	1 : 1	9 : 1	9 : 1	NA	9 : 1	1 : 1
*w*_hw_ : *w*_hp_	NA	1 : 9	NA	9 : 1	1 : 1	1 : 9	1 : 9	NA	9 : 1	1 : 1

**Table 8:** The number of effective MT systems in WMT13.

Year	Number of evaluated MT systems									
	Other-to-English					English-to-other				
	CS-EN	DE-EN	ES-EN	FR-EN	RU-EN	EN-CS	EN-DE	EN-ES	EN-FR	EN-RU
WMT13	12	23	17	19	23	14	21	18	23	19

**Table 9:** The performances on WMT13 shared tasks using Spearman rank correlation.

System	Correlation score with human judgments											
	Other-to-English						English-to-other					
	CS-EN	DE-EN	ES-EN	FR-EN	RU-EN	Mean	EN-CS	EN-DE	EN-ES	EN-FR	EN-RU	Mean
hLEPOR	0.80	0.93	0.75	0.95	0.79	0.84	0.75	**0.90**	0.84	0.90	**0.85**	**0.85**
nLEPOR_*A*_	0.85	0.95	0.83	0.95	0.72	0.86	0.82	**0.90**	**0.85**	**0.92**	0.73	0.84
METEOR	**0.96**	**0.96**	**0.98**	0.98	**0.81**	**0.94**	**0.94**	0.88	0.78	**0.92**	0.57	0.82
BLEU	0.94	0.90	0.88	**0.99**	0.67	0.88	0.90	0.79	0.76	0.90	0.57	0.78
TER	0.80	0.83	0.83	0.95	0.60	0.80	0.86	0.85	0.75	0.91	0.54	0.78

Bold fonts mean the best performance.

**Table 10:** The performances on WMT13 shared tasks using Pearson correlation.

System	Correlation score with human judgments											
	Other-to-English						English-to-other					
	CS-EN	DE-EN	ES-EN	FR-EN	RU-EN	Mean	EN-CS	EN-DE	EN-ES	EN-FR	EN-RU	Mean
hLEPOR	0.81	0.96	0.90	0.96	0.71	0.87	0.76	**0.94**	**0.91**	0.91	**0.77**	**0.86**
nLEPOR_*A*_	0.80	0.94	0.94	0.96	0.69	0.87	**0.82**	0.92	0.90	**0.92**	0.68	0.85
METEOR	**0.99**	**0.96**	**0.97**	**0.98**	**0.84**	**0.95**	**0.82**	0.88	0.88	0.91	0.55	0.81
BLEU	0.89	0.91	0.94	0.94	0.60	0.86	0.80	0.82	0.88	0.90	0.62	0.80
TER	0.77	0.87	0.91	0.93	0.80	0.80	0.70	0.73	0.78	0.91	0.61	0.75

Bold fonts mean the best performance.

**Table 11:** 

POS	Frequency
**$∗LRB∗ **	213
$,	4590
$.	3270
**∗T1∗ **	68
**∗T2∗ **	11
**∗T3∗ **	3
**∗T4∗ **	1
ADJA	4337
ADJD	1943
ADV	2900
APPO	14
APPR	5325
APPRART	1187
APZR	29
ART	7748
CARD	1135
FM	172
ITJ	1
KOKOM	166
KON	1943
KOUI	136
KOUS	929
NE	4102
NN	15211
PDAT	340
PDS	292
PIAT	252
**PIDAT**	292
PIS	824
PPER	1827
PPOSAT	703
PRELAT	38
PRELS	810
**PRF **	471
**PROAV **	280
PTKA	37
PTKANT	5
PTKNEG	393
PTKVZ	433
PTKZU	476
**PWAT **	14
**PWAV **	240
**PWS **	86
TRUNC	18
VAFIN	2197
VAINF	202
VAPP	84
VMFIN	417
VMINF	4
VVFIN	3818
VVIMP	18
VVINF	1051
VVIZU	173
VVPP	1408
XY	14

## Appendix

This appendix offers the POS tags and their occurrences number in one of WMT2012 German documents containing 3,003 sentences and parsed by Berkeley parser for German language, that is, trained on German Negra Treebank.

Number of different POS tags in the document is 55. Detailed POS labels (boldface POSs are not covered in the original mapping) and their frequencies are given in Table 11.

## References

[1] Weaver W, Locke W, Donald Booth A (1955). Translation. *Machine Translation of Languages: Fourteen Essays*.

[2] Och FJ Minimum error rate training for statistical machine translation.

[3] Marino JB, Banchs RE, Crego JM (2006). N-gram-based machine translation. *Computational Linguistics*.

[4] Lembersky G, Ordan N, Wintner S (2012). Language models for machine translation-original vs. translated texts. *Journal of Computational Linguistics*.

[5] Papineni K, Roukos S, Ward T, Zhu WJ BLEU: a method for automatic evaluation of machine translation.

[6] Banerjee S, Lavie A METE-OR: an Automatic Metric for MT Evaluation with Improved Correlation with Human Judgments.

[7] Snover M, Dorr B, Schwartz R, Micciulla L, Makhoul J A study of translation edit rate with targeted human annotation.

[8] Chen B, Kuhn R Amber: a modi-fied bleu, enhanced ranking metric.

[9] Zhang Y, Vogel S, Waibel A Interpreting BLEU/NIST scores: how much improvement do we need to have a better system?.

[10] Denkowski M, Lavie A Meteor 1.3: automatic metric for reliable optimization and evaluation of machine translation systems.

[11] Chen B, Kuhn R, Foster G Improving AMBER, an MT evaluation metric.

[12] Doddington G Automatic evaluation of machine translation quality using n-gram co-occurrence statistics.

[13] Wong B, Kit C (2009). ATEC: automatic evaluation of machine translation via word choice and word order. *Machine Translation*.

[14] Han ALF, Wong DF, Chao LS LEPOR: a robust evaluation metric for machine translation with augmented factors.

[15] Han ALF, Wong DF, Chao LS Language-independent model for machine translation evaluation with reinforced factors.

[16] Han ALF, Wong DF, Chao LS, He L, Li S, Zhu L (2013). Phrase tagset mapping for French and English treebanks and its application in machine translation evaluation. *Language Processing and Knowledge in the Web*.

[17] Han ALF, Lu Y, Wong DF, Chao LS, He L, Xing J Quality estimation for machine translation using the joint method of evaluation criteria and statistical modeling.

[18] Gamon M, Aue A, Smets M Sentence-level MT evaluation without reference translations: beyond language modeling.

[19] Albrecht JS, Hwa R Regression for sentence-level MT evaluation with pseudo references.

[20] Specia L, Giménez J Combining confidence estimation and reference-based metrics for segment-level MT evaluation.

[21] Specia L, Raj D, Turchi M (2010). Machine translation evaluation versus quality estimation. *Machine Translation*.

[22] Popović M, Vilar D, Avramidis E, Burchardt A Evaluation without references: IBM1 scores as evaluation metrics.

[23] Mehdad Y, Negri M, Federico M Match without a referee: evaluating MT adequacy without reference translations.

[24] Avramidis E Comparative quality estimation: automatic sentence-level ranking of multiple machine translation outputs.

[25] Callison-Burch C, Koehn P, Monz C, Post M, Soricut R, Specia L Findings of the 2012 workshop on statistical machine translation.

[26] Felice M, Specia L Linguistic features for quality estimation.

[27] Petrov S, Das D, McDonald R A universal part-of-speech tagset.

[28] Marcus M, Santorini B, Marcinkiewicz MA (1993). Building a large annotated corpus of English: the Penn Treebank. *Computational Linguistics*.

[29] Skut W, Krenn B, Brants T, Uszkoreit H An annotation scheme for free word order languages.

[30] Petrov S, Barrett L, Thibaux R, Klein D Learning accurate, compact, and interpretable tree annotation.

[31] Macháček M, Bojar O Results of the WMT13 metrics shared task.

[32] Callison-Burch C, Koehn P, Monz C, Zaidan OF Findings of the 2011 workshop on statistical machine translation.

